# Tendências de Busca na Internet e Tendências de Mortalidade Regional: O Caso de Anticoagulantes Orais e Acidente Vascular Cerebral

**DOI:** 10.36660/abc.20190768

**Published:** 2020-05-12

**Authors:** Roberto Muniz Ferreira, Ísis da Capela Pinheiro, João Roquette Fleury da Rocha

**Affiliations:** 1 Universidade Federal do Rio de Janeiro Instituto do Coração Édson Saad Rio de Janeiro RJ Brasil Universidade Federal do Rio de Janeiro – Instituto do Coração Édson Saad,Rio de Janeiro, RJ – Brasil; 2 Hospital Samaritano Botafogo Rio de Janeiro RJ Brasil Hospital Samaritano Botafogo – Cardiologia,Rio de Janeiro, RJ – Brasil

**Keywords:** Acidente Vascular Cerebral/prevenção e controle, Isquemia Miocárdica/prevenção e controle, Disponibilidade de Medicamentos via Internet/tendências, Anticoagulantes/uso terapêutico, Varfarina, Dabigatrana, Rivaroxabana, Mortalidade/tendências

## Introdução

Vários estudos relataram que até 30% dos casos de acidente vascular cerebral (AVC) isquêmico estão associados com fibrilação atrial (FA), especialmente na população idosa.^1 , 2^ Apesar de o risco de embolia variar conforme características clínicas e comorbidades, a terapia anticoagulante tem mostrado consistentemente uma redução nas taxas de AVC de aproximadamente 70%.^1^ No entanto, estudos prévios mostraram que as taxas de tratamento com varfarina eram baixas, mesmo em pacientes com risco elevado para eventos. Apesar da evidência de várias publicações mostrando a eficácia e a segurança dos antagonistas da vitamina K, sua complexa farmacocinética e a necessidade de monitoramento contínuo e ajustes frequentes da dosagem foram as principais justificativas para baixa adesão.^3^

Durante os últimos 10 anos, quatro Anticoagulantes Orais Diretos (AODs) tornaram-se disponíveis para prevenir eventos embólicos em pacientes com FA não valvular: dabigatrana, rivaroxabana, apixabana e edoxabana. Em agosto de 2011, a dabigatrana foi o primeiro AOD aprovado no Brasil para prevenção de AVC, seguido da rivaroxabana quatro meses depois. Dois anos depois, a apixabana também foi introduzida no mercado brasileiro e somente em fevereiro de 2018 a edoxabana se tornou disponível. Compara à varfarina, muitos ensaios sugeriram que os AODs não são inferiores na prevenção de AVCs isquêmicos e possivelmente superiores na redução da mortalidade, talvez devido à menor ocorrência de hemorragias intracranianas.^4^ Além de não necessitar monitoramento laboratorial, os AODs têm uma farmacocinética mais previsível e uma incidência mais baixa de interações medicamentosas. Estudos recentes demonstraram um aumento na taxa de prescrições de anticoagulantes entre os médicos desde que os AODs se tornaram disponíveis na prática clínica.^1^

Análise de tendência de busca na Internet é um método promissor para estimar a frequência com que intervenções médicas têm sido aplicadas na prática clínica. Publicações mais recentes sugeriram uma forte correlação entre os termos inseridos nas buscas na rede, decisões médicas e padrões de prescrição farmacológica para uma dada região.^5^ No entanto, não está claro se esses padrões de busca também são preditivos de tendências regionais associadas a ventos clínicos.

## Tendências de busca na Internet na assistência à saúde

Atualmente, o Google talvez seja a ferramenta de busca mais utilizada, mesmo entre os profissionais de saúde. Os padrões de busca criados dentro do Google estão disponíveis desde 2004 e podem ser acessados a partir do Google Trends (Google Inc. Mountain View, CA, EUA). Em resumo, trata-se de uma ferramenta de acesso aberto que apresenta com que frequência um dado termo ou tópico foi buscado no programa de busca Google. Além disso, podem ser aplicados filtros para especificar uma região e o período para a análise. A frequência á apresentada como um número entre 0 e 100, que varia ao longo do intervalo de tempo pré-definido e representa uma proporção em relação ao ponto mais alto de popularidade. Assim, um valor de 100 indica o momento em que o termo ou tópico alcançou o interesse mais alto de busca, e um valor de zero correlaciona-se com menos que 1% da popularidade máxima.^6^ Além disso, até cinco termos podem ser analisados simultaneamente, e um valor médio de popularidade é automaticamente gerado para cada termo durante o intervalo selecionado.

Os sistemas de busca de Internet têm o potencial de refletir o interesse geral de uma população sobre um dado tópico, dentro de um intervalo de tempo e uma região específicos. O Google Trends é um exemplo dessa ferramenta, e seus escores resultam de muitos fatores que influenciam diretamente o entendimento do público acerca do assunto pesquisado. Esses fatores incluem campanhas promocionais, cobertura da mídia, taxas de alfabetização e status socioeconômico. No entanto, quando pacientes e profissionais de saúde são expostos à informação e conhecimento, existe uma maior probabilidade de uma tomada de decisão informada em relação à implementação de intervenções médicas.

Um estudo de Kritz et al.,^7^ mostrou que os médicos utilizam frequentemente sistemas gerais de busca para obterem conhecimento médico na prática diária, principalmente pela falta de tempo para uma pesquisa mais ampla.^7^ Além disso, alguns países utilizam sistemas de busca como ferramentas de pesquisa epidemiológica para uma variedade de doenças, o que poderia ter implicações nas políticas públicas de saúde. Na França, o Sentinel Network é um sistema público de monitoramento de saúde pública em que médicos clínicos gerais utilizam dados baseados em rede para acompanharem padrões de doenças e potencialmente identificarem surtos em estágio inicial.^8^

Apesar de escores de popularidade não necessariamente refletirem os padrões de prescrição de medicamentos, estudos anteriores incluindo uma ampla variedade de medicamentos sugeriram que uma associação de fato existe. Essa associação foi demonstrada com estatinas e vários medicamentos não cardiovasculares.^9 , 10^ Um estudo de Lippi et al.,^6^ também apontou um aumento no volume de busca online por AODs, o que condiz com o rápido aumento na prática clínica.

## Tendências de busca na Internet para anticoagulantes orais e AVC

Apesar do aumento progressivo no número de publicações nessa área, a associação entre padrões específicos de busca de tratamento e variações subsequentes nos eventos clínicos populacionais necessita ainda ser demonstrada. Se uma relação realmente existe, os dados de busca poderiam funcionar como um substituto para efeitos em grande escala de um dado tratamento farmacológico ou intervenção em relação a desfechos clínicos específicos. A influência de anticoagulantes orais na epidemiologia de mortes relacionadas a AVC serve como um exemplo adequado nesse cenário, considerando que a maioria dos eventos cerebrovasculares isquêmicos são cardioembólicos e prevenidos por anticoagulantes orais. Hernandez et al.,^11^ correlacionaram a variação geográfica no uso de anticoagulantes e as taxas de AVC em usuários do Medicare, demonstrando uma relação inversa entre as duas variáveis.^11^

Segundo o departamento de tecnologia da informação do Sistema Único de Saúde (DATASUS), o número total de mortes relacionadas a AVC no Brasil diminuiu de 2006 a 2015, apesar que a redução mais significativa tenha ocorrido após 2011.^12^ Além disso, AVC isquêmico e os casos de AVC sem classificação específica (ICD-10 códigos I63 e I64) foram a maioria dos eventos cerebrovasculares (70.2%), e apresentaram um padrão similar de declínio após 2011 (média de 49 406,4 ± 451 vs. 46447,2 ± 1633 de óbitos por ano antes e após 2011, respectivamente). Por outro lado, mortes por AVC hemorrágico (ICD-10 códigos I60, I61 e I62) aumentaram no mesmo período, apesar que em uma proporção bem menor (média de 19 740,4 ± 278 vs. 20 933.8 ± 446 de óbitos por ano antes e após 2011, respectivamente).^13^

Durante o mesmo período, quando varfarina, dabigatrana e rivaroxabana foram usadas como tópicos de busca, e o Brasil como a única região de busca, observou-se um franco declínio nos escores do Google Trands para varfarina após 2011. Em 2015, o valor de popularidade havia atingido aproximadamente o mesmo nível que em 2009. Por outro lado, o score para rivaroxabana aumentou consideravelmente após 2011, e ultrapassou a popularidade da varfarina após 2013. O escore de busca para dabigarana permaneceu consistentemente abaixo dos outros dois anticoagulantes por todo o período de tempo analisado. Deve-se ressaltar que, quando um tópico (ou seja, “medicamento”) é usado como opção de busca, os termos associados ao medicamento, incluindo nomes comerciais, também são contemplados.

**Figura 1:**
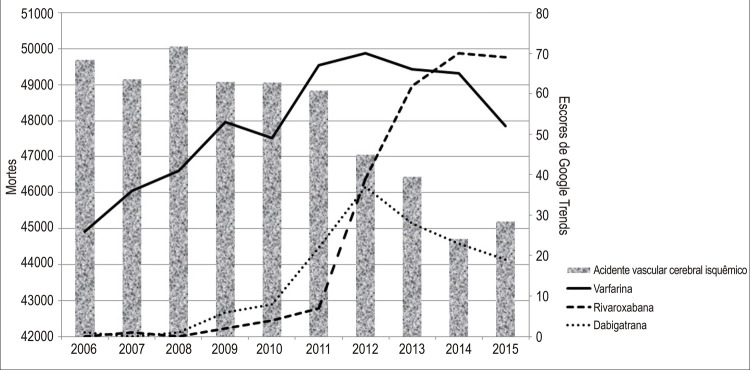
– *Mortes por acidente vascular cerebral isquêmico por ano e média de escores do Google Trends para anticoagulantes. Após 2011, houve um aumento na popularidade online da rivaroxabana acompanhada por um decréscimo no número total de mortes relacionadas a acidente vascular cerebral isquêmico no Brasil.*

**Figura 2:**
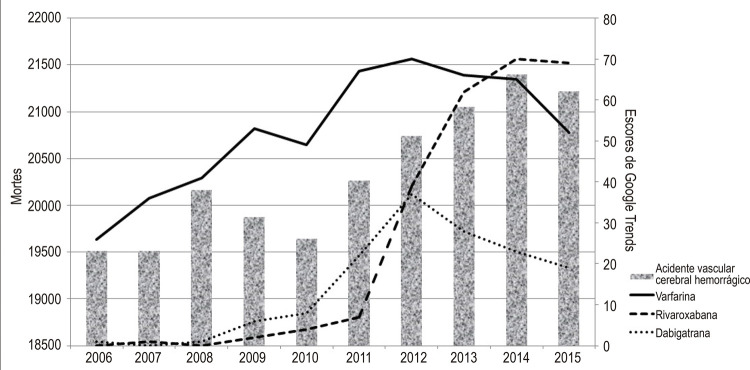
– *Mortes por acidente vascular cerebral hemorrágico por ano e média de escores do Google Trends para anticoagulantes. Após 2011, houve um aumento na popularidade online da rivaroxabana acompanhada por um aumento escalonado no número de mortes relacionadas a acidente vascular cerebral hemorrágico no Brasil.*

Quando as mortes relacionadas a AVC e escores de busca são analisados em combinação, parece existir uma correlação inversa com AVC isquêmico e uma associação positiva com eventos hemorrágicos. Entre 2011 e 2015, mortalidade total e por AVC isquêmico diminuiu ( Figura 1 ), ao passo que eventos hemorrágicos aumentaram ( Figura 2 ) concomitantemente ao aumento dos escores do Google Trends para AODs. Mais importante, essa relação parece ter sido primariamente causada por um aumento da popularidade da rivaroxabana.

Uma vez que tem ocorrido um aumento progressivo na prescrição de anticoagulantes orais na prática clínica, além de um aumento na popularidade dos AODs na Internet em todo o mundo, modificações na incidência de eventos cerebrovasculares isquêmicos e hemorrágicos podem ser antecipadas.^1 , 6^ No Brasil, tal impacto também seria esperado, já que desde 2015 a rivaroxabana já era a droga de maior receita de venda no mercado nacional, apenas quatro anos após ter se tornado disponível ao público.^14^ Em apenas dois anos, a rivaroxabana ultrapassou a varfarina em volume de busca na Internet na maior parte do país. Esses achados provavelmente se devem a um aumento no uso de AODs, em vez de uma transição entre categorias de anticoagulantes. Os AODs tornaram-se opções atrativas quando a prevenção de AVC é considerada em FA, principalmente devido à sua farmacocinética previsível, provavelmente maior segurança e eficácia não inferior à de antagonistas da vitamina K.

Ao contrário do que foi encontrado em relação a AVC isquêmico, mortes relacionadas a AVC hemorrágico aparentemente aumentaram desde 2011. Apesar de tal aumento haver ocorrido em uma taxa mais lenta, a tendência parece também estar relacionada aos padrões de busca por rivaroxabana. Talvez essa tendência tenha sido consequente ao grande número de pacientes sem tratamento prévio que progressivamente receberam anticoagulantes, já que os AODs tendem a reduzir sangramento intracraniano em comparação à varfarina.

A maior utilização do escore CHA_2_DS_2_-Vasc Score também pode ter contribuído para o aumento no número de pacientes recebendo terapia anticoagulante durante o período de estudo.^1^ Além disso, considerando que a mortalidade total por AVC diminuiu significativamente, o padrão epidemiológico é comparável ao benefício líquido dos AODs descrito em muitos ensaios.^4^ A possibilidade de que outros fatores, tais como políticas de saúde pública, maior controle dos fatores de risco cardiovascular, e melhores condições socioeconômicas, possa ter influenciado o número anual de mortes não pode ser excluída. Contudo, uma redução nas mortes relacionadas a AVC hemorrágico também já seria esperada como resultado dessas intervenções. Até 2017, observou-se um declínio consistente nas mortes isquêmicas, enquanto os eventos hemorrágicos continuaram a aumentar.^12^

Apesar de os escores de busca fornecerem uma estimativa dos padrões de prescrição, não são um reflexo direto de utilização ou vendas regionais dos medicamentos. O acesso à Internet em 2014 estava disponível em aproximadamente 50% dos domicílios no Brasil e a taxa de analfabetismo em indivíduos acima de 65 anos ainda era alta (26,4%).^15^ Assim, esses padrões devem ser interpretados considerando todos os potenciais vieses, principalmente pelo fato de os algoritmos específicos empregados pelo Google Trends não terem sido apresentados. Porém, uma vez que o acesso à Internet aumenta em todo o mundo, e novas políticas são desenvolvidas para a redução das taxas de analfabetismo, as tendências de busca se tornarão cada vez mais correlacionados a padrões diários de comportamento.

## Conclusão

O progressivo crescimento da população mundial tem demandado o desenvolvimento de novos mecanismos para monitorar mudanças epidemiológicas tanto nas tendências de tratamento como nos padrões de doença. Nesse contexto, a Internet tornou-se uma ferramenta valiosa para reunir informações na tomada de decisão diária, particularmente no cuidado à saúde, em que a análise crítica dos dados coletados também é de suma importância. Nos últimos dez anos, o aumento em experiência clínica com AODs em pacientes com FA foi acompanhado por um aumento global significativo na popularidade dessas drogas em sistemas de busca na Internet. Esse fenômeno parece também estar ocorrendo em países de renda média, tal como o Brasil. No entanto, a associação entre tendências de buscas na rede e desfechos clínicos continua uma área pouco estudada. Existe a possibilidade de que a eficácia de políticas de saúde e intervenções em grande escala, tais como campanhas de vacinação, possam ser monitoradas por dados de busca online, principalmente em regiões onde a maioria da população tem acesso à Internet. Áreas específicas da medicina onde essa estratégia possa ser valiosa precisam ser determinadas e exploradas em estudos futuros.

## References

[1] Katz D, Maddox T, Turakhia M, Gehi A, O’Brien E, Lubitz S (2017). Contemporary Trends in Oral Anticoagulant Prescription in Atrial Fibrillation Patients at Low to Moderate Risk of Stroke After Guideline-Recommended Change in Use of the CHADS 2 to the CHA 2 DS 2 -VASc Score for Thromboembolic Risk Assessment. Circ Cardiovasc Qual Outcomes.

[2] Kirchhof P, Benussi S, Kotecha D, Ahlsson A, Atar D, Casadei B (2016). 2016 ESC Guidelines for the management of atrial fibrillation developed in collaboration with EACTS. Eur Heart J.

[3] Buckingham T, Hatala R (2002). Anticoagulants for atrial fibrillation: Why is the treatment rate so low?. Clin Cardiol.

[4] López-López J, Sterne J, Thom H, Higgins J, Hingorani A, Okoli J (2017). Oral anticoagulants for prevention of stroke in atrial fibrillation: systematic review, network meta-analysis, and cost effectiveness analysis. BMJ.

[5] Moat H, Olivola C, Chater N, Preis T (2016). Searching Choices: Quantifying Decision-Making Processes Using Search Engine Data. Top Cogn Sci.

[6] Lippi G, Mattiuzzi C, Cervellin G, Favaloro E (2017). Direct oral anticoagulants: analysis of worldwide use and popularity using Google Trends. Ann Transl Med.

[7] Kritz M, Gschwandtner M, Stefanov V, Hanbury A, Samwald M (2013). Utilization and Perceived Problems of Online Medical Resources and Search Tools Among Different Groups of European Physicians. J Med Internet Res.

[8] Blanchon T, Nkuchia M, Lynfield R, Van Beneden CA, de Valk H (2013). Infectious dsease surveillance.

[9] Simmering J, Polgreen L, Polgreen P (2014). Web search query volume as a measure of pharmaceutical utilization and changes in prescribing patterns. Res Social Adm Pharm.

[10] Schuster N, Rogers M, McMahon L (2010). Using Search Engine Query Data to Track Pharmaceutical Utilization: A Study of Statins. Am J Manag Care.

[11] Hernandez I., Saba S., Zhang Y (2017). Geographic Variation in the Use of Oral Anticoagulation Therapy in Stroke Prevention in Atrial Fibrillation. Stroke.

[12] Brasil, Ministério da Saúde, Datasus (2018). Informações estatísticas da saúde.

[13] World Health Organization . (WHO) (2018). International Classification of Diseases.

[14] Interfarma, Associação da Indústria Farmacêutica de Pesquisa (2016). Guia 2016 Interfarma.

[15] Instituto Brasileiro de Geografia e Estatística. (IBGE) (2018). Indicadores 2015.

