# Supplementary material for: Tendências de Busca na Internet e Tendências de Mortalidade Regional: O Caso de Anticoagulantes Orais e Acidente Vascular Cerebral
Source: Arq Bras Cardiol. 2020 May 12;114(4):726–9. [Article in Portuguese] doi: 10.36660/abc.20190768 (PMC9744337; doi:10.36660/abc.20190768)
Supplement: Translation

# Translation: Internet Search Trends and Regional Mortality Tendencies: The Case of Oral Anticoagulants and Stroke

**Authors:** Roberto Muniz Ferreira, Ísis da Capela Pinheiro, João Roquette Fleury da Rocha

**Affiliations:** 1 Brazil Universidade Federal do Rio de Janeiro – Instituto do Coração Édson Saad, Rio de Janeiro, RJ – Brazil; 2 Brazil Hospital Samaritano Botafogo – Cardiologia, Rio de Janeiro, RJ – Brazil

**Keywords:** Stroke/prevention and control, Myocardial Ischemia/prevention and control, Pharmaceutical Services, Online/trends, Anticoagulants/therapeutic use, Varfarin, Dabigatran, Rivaroxaban, Mortality/trends

## Introduction

Multiple studies have found that up to 30% of ischemic strokes are associated with atrial fibrillation (AF), especially in the elderly population.^1 , 2^ Although the embolic risk varies according to clinical characteristics and comorbidities, anticoagulant therapy has consistently shown to reduce stroke rates by approximately 70%.^1^ However, previous studies demonstrated that treatment rates with warfarin were low, even in patients at high risk for embolic events. Despite evidence from various publications demonstrating the effectiveness and safety of vitamin K antagonists, their complex pharmacokinetics and the need for continuous monitoring and frequent dose adjustments were the main explanations for low adherence.^3^

Over the last 10 years, four direct oral anticoagulants (DOAC) have become available to prevent embolic events in patients with non-valvular AF: dabigatran, rivaroxaban, apixaban and edoxaban. In August 2011, dabigatran was the first DOAC approved in Brazil for stroke prevention, followed by rivaroxaban four months later. Two years later, apixaban was also introduced in the Brazilian market and only in February 2018 edoxaban became available. When compared to warfarin, multiple trials have suggested that DOACs are non-inferior in preventing ischemic strokes and possibly superior in reducing mortality, perhaps due to fewer intracranial hemorrhages.^4^ In addition to waive the need for laboratory monitoring, they have more predictable pharmacokinetics and a lower incidence of drug-to-drug interactions. Recent studies have demonstrated an increase in anticoagulation prescription rates among physicians since DOACs have become clinically available.^1^

The analysis of Internet search trends is a promising method for estimating the frequency by which medical interventions are being applied in clinical practice. More recent publications have suggested a strong correlation between Internet search engine query data, medical decision making and pharmacological prescription patterns for a given region.^5^ However, it is not clear whether these search patterns are also predictive of regional trends associated with clinical events.

## Internet search trends in health care

Currently, Google is perhaps the most utilized online search tool, even among healthcare professionals. The search patterns created within Google have been available since 2004 and can be accessed from Google Trends (Google Inc. Mountain View, CA, USA). Briefly, this is an open-access tool which displays how frequently any given term or topic has been searched for in the Google search engine. Additionally, filters can be used to specify a region and time period for the analysis. The frequency is presented as a number from 0 to 100 which varies over the predefined time interval and represents a proportion in relation to the highest popularity point. Accordingly, a value of 100 indicates the moment at which the term or topic reached the highest search interest, and a value of zero correlates to less than 1% of the peak popularity.^6^ Also, up to five terms or topics can be analyzed simultaneously, and a mean popularity value is automatically provided for each term during the selected interval.

Internet search engines have the potential to reflect the general interest of a population in a given topic, within a specific time interval and region. Google Trends is an example of such a tool, and the scores provided the website are a result of many factors that directly influence the public’s awareness regarding the subject being researched. These include promotional campaigns, media coverage, internet access, literacy rates and socioeconomic status. Nevertheless, when patients and health care professionals are exposed to information and knowledge, there is a higher probability of an informed decision regarding the implementation of medical interventions.

A study by Kritz et al.^7^ demonstrated that physicians frequently use general search engines to retrieve medical knowledge in daily practice, chiefly because of lack of time for a more thorough research.^7^ Furthermore, some countries use search engines as epidemiological surveillance tools for a variety of diseases, which could have implications in public health policies. In France, the Sentinel Network is a public health monitoring system where general practitioners use web-based data to follow disease patterns and potentially identify outbreaks at an early stage.^8^

Although popularity scores do not necessarily mirror drug prescription patterns, previous studies with a wide variety of medications have suggested that an association does in fact exist. This association has been shown with statins and several non-cardiovascular drugs.^9 , 10^ A previous study by Lippi et al.^6^ also found an increase in worldwide online search volume for DOACs, which has been consistent with the escalation in clinical use.

## Internet search trends for oral anticoagulants and stroke

Despite a progressive increase in the number of publications in this area, an association between specific patterns of treatment search and subsequent variations in populational clinical events has yet to be clearly demonstrated. If a relationship really exists, search data would have the potential to function as a surrogate for large-scale effects of a given drug or intervention regarding specific clinical outcomes. The influence of oral anticoagulants in the epidemiology of stroke-related deaths serves as an adequate example in this scenario, considering that most ischemic cerebrovascular events are cardioembolic and effectively prevented by oral anticoagulants. Hernandez et al.^11^ have previously correlated geographic variation in the use of anticoagulation with stroke rates among Medicare beneficiaries, demonstrating an inverse relationship between the two variables.^11^

According to the Department of Information Technology of the Brazilian Unified Health System (DATASUS), the total number of stroke-related deaths in Brazil declined between 2006 and 2015, although the most significant reduction was after 2011.^12^ In addition, ischemic strokes and those not classified as a specific type (ICD-10 codes I63 and I64) comprised most of the cerebrovascular events (70,2%), and presented a similar pattern of decline since 2011 (mean of 49,406.4 ± 451 vs. 46,447.2 ± 1633 deaths per year before and after 2011, respectively). Conversely, hemorrhagic stroke deaths (ICD-10 codes I60, I61 and I62) increased in the same period, albeit at a much lower proportion (mean of 19,740.4 ± 278 vs 20,933.8 ± 446 deaths per year before and after 2011, respectively).^13^

During the same period, when warfarin, dabigatran and rivaroxaban were used as search topics (“drug”) and Brazil was defined as the only search region, there was a clear tendency for a decline in warfarin’s Google Trends search scores after 2011. In 2015 the popularity value reached approximately the same level as 2009. Conversely, rivaroxaban’s score increased considerably after 2011, and surpassed warfarin’s popularity after 2013. Dabigatran’s search score remained consistently below the other two anticoagulants throughout the analyzed time interval. It should be noted that when a topic (i.e. “drug”) is used as the search option, terms that are associated with the corresponding drug, including commercial names, are also contemplated.

When stroke-related deaths and search scores are appreciated in combination there appears to be an inverse correlation with ischemic strokes and a positive association with hemorrhagic events. Between 2011 and 2015, total and ischemic stroke deaths decreased ( Figure 1 ) and hemorrhagic events increased ( Figure 2 ) concurrently to an escalation in DOAC Google Trends’ search scores. Most importantly, this relationship seemed to be primarily driven by a rise in rivaroxaban’s popularity.

**Figure 1:** – *Annual ischemic stroke-related deaths and mean Google Trends scores for anticoagulants. After 2011, an increase in the online popularity of rivaroxaban was accompanied by a decrease in the number of ischemic stroke related-deaths in Brazil.*

**Figure 2:** – *Annual hemorrhagic stroke-related deaths and mean anticoagulant Google Trends scores. After 2011, an increase in rivaroxaban’s online popularity was accompanied by an escalation in the total number of hemorrhagic stroke related-deaths in Brazil.*

Since there has been a progressive increase in the prescription of oral anticoagulants in clinical practice, and also a rise in the internet popularity of DOAC worldwide, changes in the incidence of both ischemic and hemorrhagic cerebrovascular events may be anticipated.^1 , 6^ In Brazil, such an impact would also be expected, since in 2015 rivaroxaban was already the drug with the third highest sales revenue in the national market, only four years after it became available to the public.^14^ In only two years, rivaroxaban surpassed warfarin in Internet search volume in most of the country. This finding is probably related to an increase in the use of DOACs, rather than a transition between anticoagulant categories. DOACs have become attractive options when stroke prevention is considered in AF, mainly because of their predictable pharmacokinetics, probably safer profile and non-inferior effectiveness when compared to vitamin K antagonists.

Contrary to what was found with ischemic strokes, hemorrhagic stroke-related deaths apparently increased since 2011. Although such an escalation occurred at a lower rate, the trend also appeared to be related to rivaroxaban´s search patterns. Perhaps this tendency was also a result of a greater number of previously untreated patients that progressively received anticoagulants, since DOACs tend to reduce intracranial bleeding when compared to warfarin.

The greater adoption of the CHA_2_DS_2_-Vasc Score could also have contributed in expanding the number of patients on anticoagulant therapy during the study period.^1^ Furthermore, considering that total stroke deaths decreased significantly, the epidemiological pattern is comparable to the net benefit of DOACs that has been found in multiple trials.^4^ The possibility that other factors, such as public health policies, greater cardiovascular risk factor control and improvements in socioeconomic conditions, may have influenced the annual number of deaths cannot be entirely excluded. However, a reduction in hemorrhagic stroke-related deaths would be expected solely from these interventions. Until 2017, a persistent decline in ischemic deaths was still observed, whereas hemorrhagic events continued to increase.^12^

Although search scores may provide an estimate of prescription patterns, they are not a direct reflection of regional drug utilization or sales. Internet access in 2014 was available in approximately 50% of all households in Brazil and the illiteracy rate in individuals over 65 years of age was still high (26.4%).^15^ As such, these patterns must be interpreted considering all of the potential biases, especially because the specific algorithms that were employed by Google Trends were not disclosed. Nevertheless, as Internet access expands throughout the World and new policies are developed to reduce illiteracy rates, search trends will become increasingly more correlated to daily behavioral patterns.

## Conclusion

The progressive worldwide populational growth has demanded the development of new mechanisms to monitor epidemiological changes in both treatment tendencies and disease patterns. In this context, the Internet has become a valuable tool for gathering information to aid in daily decision making, particularly in health care, where the critical appraisal of the collected data is also of upmost importance. Over the last 10 years, the increasing clinical experience with DOACs in patients with AF has been accompanied by a significant global rise in the popularity of these drugs in Internet search engines. This phenomenon also appears to be occurring in middle-income countries, such as Brazil. However, the association between web-based tendencies and clinical outcomes is still an area that needs further investigation. There is a possibility that the effectiveness of large-scale health care policies and interventions, such as vaccination campaigns, may be monitored by online search data, especially in regions where most of the population have access to the internet. Specific areas in medicine where this strategy may be of value are yet to be determined and should be explored in future studies.
